# Degradation of phenol via ortho-pathway by *Kocuria sp*. strain TIBETAN4 isolated from the soils around Qinghai Lake in China

**DOI:** 10.1371/journal.pone.0199572

**Published:** 2018-06-27

**Authors:** Leyang Wu, Daniel C. Ali, Peng Liu, Cheng Peng, Jingxin Zhai, Ying Wang, Boping Ye

**Affiliations:** School of Life Science and Technology, China Pharmaceutical University, Nanjing, People’s Republic of China; Babasaheb Bhimrao Ambedkar University, INDIA

## Abstract

Based on the feature of high-altitude permafrost topography and the diverse microbial ecological communities of the Qinghai-Tibetan Plateau, soil samples from thirteen different collection points around Qinghai lake were collected to screen for extremophilic strains with the ability to degrade phenol, and one bacterial strain recorded as TIBETAN4 that showed effective biodegradation of phenol was isolated and identified. TIBETAN4 was closely related to *Kocuria* based on its observed morphological, molecular and biochemical characteristics. TIBETAN4 grew well in the LB medium at pH 7–9 and 0–4% NaCl showing alkalophilicity and halophilism. The isolate could also tolerate up to 12.5 mM phenol and could degrade 5 mM phenol within 3 days. It maintained a high phenol degradation rate at pH 7–9 and 0–3% NaCl in MSM with 5 mM phenol added as the sole carbon source. Moreover, TIBETAN4 could maintain efficient phenol degradation activity in MSM supplemented with both phenol and glucose and complex water environments, including co-culture *Penicillium* strains or selection of non-sterilized natural lake water as a culture. It was found that TIBETAN4 showed enzymatic activity of phenol hydroxylase and catechol 1,2-dioxygenase after induction by phenol and the corresponding genes of the two enzymes were detected in the genome of the isolate, while catechol 2,3-dioxygenase or its gene was not, which means there could be a degradation pathway of phenol through the ortho-pathway. The Q-PCR results showed that the transcripts of both the phenol hydroxylase gene and catechol 1,2-dioxygenase gene were up-regulated under the stimulation of phenol, demonstrating again that the strain degraded phenol via ortho-degradation pathway.

## Introduction

Phenol is widely distributed as an environmental pollutant due to its common presence in the effluents of many industrial sources, including oil refineries, petrochemical plants, ceramic plants, steel plants, coal conversion processes and phenolic resin industries[1], and it is frequently described as causing great harm to human health and ecological environments due to its complex and stable chemical structure[2]; additionally, it is soluble, accumulates easily in water and has great toxicity, which could be passed through the food chain[3]. Alarmingly for the world, high concentrations of phenol have been detected in the environment (50–1500 mg/l) and even in drinking water (0.21–1130 mg/l)[4]. To control the phenol content in drinking water, the World Health Organization (WHO) stipulated that the upper limit of phenol content in industrial wastewater discharge is 1 mg/l[5]. The United States Environmental Protection Agency lists phenol as a priority pollutant and sets its concentration limit in drinking water to 1.0 × 10^−8^ M[6]. There is no doubt that how to deal effectively with the excess phenol in industrial wastewater and soil has long been a problem of great urgency in a wide range of fields.

Both physical degradation, such as nanofiltration[7], and chemical degradation, such as in situ chemical oxidation[8], are important phenol degradation techniques. However, they are limited by several disadvantages, such as the huge investment costs, complex operation, high maintenance costs, high energy consumption, difficulty in degrading low concentrations of phenol, and toxicity from by-products of phenol degradation[9]. Biological degradation has been utilized as an alternative depending upon its low associated costs and complete mineralization of the xenobiotic, such as phenol and chloronitrophenols[1,10]. However, most sources of pollution such as hospital waste and industrial effluent show extreme conditions in some respects, which led to the loss of the degradation activity of strains that originally worked in the laboratory environment. Therefore, an organism with a capacity to degrade phenol in extreme conditions would have special significance for bioremediation of pollutants in comparison to their mesophilic and neutrophilic counterparts.

The Qinghai-Tibetan Plateau (QTP) is the largest high-altitude permafrost region on earth, with 54.3% of its total area affected by permafrost[11]. The diurnal variation in the surface temperature and radiation of the QTP changes considerably because of its high altitude and large atmospheric transparency. Qinghai Lake is a highly saline (12.5 g/L salinity) and alkaline (pH 9.5) lake located in a structural intermontane depression on the northeastern corner of the QTP[12,13], and the altitude of the lake is 3,196 m above sea level. Preliminary studies have shown that microorganisms isolated from the soil, water, plants and even animals of the extreme environment, like QTP, have many special physiological functions to suit the cold, salinity and other unfavorable conditions[14]. For example, microbes distributed in the permafrost, glaciers and alpine wetland are generally psychrophilic, and those in the saline soil from the mountains and lakes are halophilic[15].

In the present study, a strain of bacteria, TIBETAN4, belongs to the genera of *Kocuria*, which was successfully isolated and identified from the soil around Qinghai Lake. The isolate showed excellent resistance to salinity and alkalinity and maintained an efficient phenol degradation activity in some harsh environments such as high salinity and alkalinity. To the best of our knowledge, this is the first phenol-degrading bacteria of this genus that exists under complex and harsh conditions isolated from extreme environments.

## Materials and methods

### Collection of samples and geochemical characterizations of the sampling site

Qinghai Lake is a hyper-saline and alkaline soda lake located in Mengyuan Hui Autonomous County of Qinghai, China (36°47′58.08″N 100°16′34.92″E), and it is known as the largest salt lake in the QTP with an area of approximately 4,583 km^2^ and a maximum depth of 32.8 m(S1 Fig). Due to its unique ecological and geological features, it is a site of interest to microbiologists for the cultivation of extremophilic microbes. Samples were collected from thirteen different collection points around Qinghai Lake based on the features of the topography and ecological communities of the QTP. The distribution and tabular representation of the geochemical characterization of the sampling site are presented (S1 Table). In the current study, soil samples collected from the alpine meadow around Qinghai Lake were used for screening for basophilic and halophilic phenol degradation isolates. Soil samples were collected in 50-ml pre-sterilized Falcon tubes. Samples were collected with a soil column depth of approximately 30 cm. Three samples were collected from each point using a diagonal method and mixed to form a compound sample. Finally, a total of thirteen compound samples of the soil were gathered. Obtained samples were stored on crushed ice, transferred to the laboratory and stored at -20°C until further processing.

### Screening for phenol-degrading strains

The soil was removed from the -20°C freezer and placed at 4°C for 1–2 h to be thawed slowly. The larger stone and plant roots were picked out, and the soil was ground using a mortar until no obvious lumps were observed. Approximately 2.5 g of each soil sample was inoculated into Erlenmeyer flasks containing 50 ml of liquid Mineral Salts Medium, MSM (3.78 g/L Na_2_HPO_4_•12H_2_O, 0.5 g/L KH_2_PO_4_, 5.0 g/L NH_4_Cl, 0.2 g/L MgSO_4_•7H_2_O, 1 ml/L Trace element solution, pH 6.8) sterilized by autoclaving and supplemented with 10 mM phenol filtered with an organic phase (0.22 μm) and finally incubated at 25°C, 150 rpm for 72 h.

The soil suspensions were removed and diluted with MSM to 10^−3^, 10^−4^ and 10^−5^ times. Then, 750 μl of each of the three kinds of diluted samples was spread on an MSM agar plate amended 5 mM phenol as a carbon source. The plates were incubated at 25°C for 2–7 days and observed daily. Colonies with good growth were inoculated on Gause's synthetic medium (20.0 g/L soluble starch, 1.0 g/L KNO_3_, 0.5 g/L K_2_HPO_4_, 0.5 g/L MgSO_4_·7H_2_O, 0.5 g/L NaCl, 0.01 g/L FeSO_4_·7H_2_O) plates for further purification of the different bacterial strains. Purified strains were transported into MSM supplemented with 3 mM phenol to obtain positive isolates and stored at 4°C until further processing.

### Tests of phenol degradation

For phenol degradation experiments, the cells were harvested by centrifugation (10,000 g, 10 min) from 10 ml of preculture in Gause's synthetic medium at 25°C, 150 rpm for 72 h, then washed three times with sterile saline. The resulting inoculum was incubated in 250-ml Erlenmeyer flasks containing 100 ml of MSM supplemented with 3.0 mM phenol as the sole carbon source and incubated at 25°C, 150 rpm for 72 h in the dark. One milliliter per 100 ml liquid MSM in an Erlenmeyer flask was collected and passed through a 0.22 μm organic phase filter every 24 h, and then, 20 μl of the supernatant was analyzed. The assays were carried out in triplicate.

The Agilent 1100 Series LC system (Agilent, USA) controlled by the Agilent Chem Station Software and fitted with an Alltech ApolloC18 column (250 mm × 4.6 mm ID) was used to detect the concentrations of phenol and metabolites. The operating conditions were as follows: room temperature; mobile phase, deionized water/acetonitrile (70:30, v/v) with a solvent flow rate of 1.0 ml/min. The ultraviolet analysis was carried out at 270 nm.

### DNA extraction, sequencing, and phylogenetic analysis

The strain was cultured in liquid Luria-Bertani Broth(LB) for 3 d at 25°C in darkness, and DNA extraction was done using UltracleanTM Microbial DNA Isolation Kit(MoBio, Solana Beach, USA) following the manufacturer’s protocol. DNA preps were stored at -20°C until they were used for PCR. 16s rRNA of the bacterium was amplified using 16s rRNA universal primer 27F-1492R, 27F (5’- AGA GTT TGA TCC TGG CTC AG -3’) and 1492R (5’- AAG GAG GTG ATC CAG CCG CA -3’). And the reaction mixtures of PCR were performed as reported[16]. Amplifications were performed in a DNA Engine® Peltier Thermal Cycler (Bio-Rad, USA). PCR products were visualized on a 1% agarose gel containing specific dyes for DNA and purified by using a DNA Gel Extraction Kit (TSINGKE, China).

The sequence of purified PCR products employed Genscript (Nanjing, China). The 16s rRNA gene sequence was analyzed using the BLAST package software (http://blast.ncbi.nlm.nih.gov/Blast.cgi). A sequence database was established using newly generated sequences and those previously published with consistency higher than 97% in GenBank. For phylogenetic analysis, the published sequences of the closely related organisms were retrieved in the FASTA format and aligned in *CLUSTAL*-W. Sequence alignment and phylogenetic analysis were conducted using the Molecular Evolutionary Genetics Analysis (MEGA) software version 5.1. Phylogenetic analysis was conducted using maximum likelihood (ML), and confidence levels in nodes were determined using 1,000-bootstrap replicates.

### Morphological and biochemical characterization

Biochemical characteristics of the phenol-degrading bacteria strain TIBETAN4 were inspected, including ONPG hydrolysis, lysine decarboxylases, ornithine decarboxylases, urease, phenylalanine deamination, methyl red, nitrate reduction, tryptophan deamination (Indole), malonate, hydrogen sulfide production, citrate utilization, Vogesproskaur test (VP), Esculin hydrolysis and carbohydrate utilization including xylose, adonitol, glucose, rhamnose, arabinose, lactose, raffinose, melibiose, saccharose, trehalose, cellobiose, oxidase, catalase, melanin and D-fructose according to previous reports[17,18]. The strain was streaked on LB plates and incubated at 25°C for 3 days. A single colony was picked for complex red single staining and gram staining to observe the microscopic morphology of the TIBETAN4 strain. The morphological features of the isolated colonies were observed employing a light-microscope, Motic BA210 (Motic, China), with a built-in digital camera using Motic images plus 2.0.

### Tolerance and phenol degradation ability of TIBETAN4

The tolerance and phenol degradation ability of TIBETAN4 were detected in different conditions. For tolerance, 1 ml of the isolate grown to exponential phase (OD_600_ 1.0) in LB liquid medium took 50 ml liquid LB with different pH levels and NaCl concentrations, and the isolate was cultured continuously at 150 rpm, 25°C for 7 days in the darkness. For phenol degradation ability, the *Kocuria* sp. strain TIBETAN4 was cultivated in 50 ml of LB at 150 rpm and 25°C until an OD_600_ value up to approximately 1.0. Prior to undertaking the inoculation, the cells were harvested by centrifugation (10,000 g, 10 min) and washed three times with the same volume of sterile normal saline. Then, 1 ml of this culture was incubated in a 250-ml Erlenmeyer flask containing 100 ml of MSM with different pH levels, different concentrations of NaCl and glucose added with 5 mM phenol. OD_600_ values and phenol concentration were measured every 24 h. To study the degradation of phenol in a complex environment, the strain was co-cultured with *Penicillium chrysogenum* CBS 306.48 (China Center of Industrial Culture Collection, CICC) in 100 ml MSM mixed with 2.5 mM phenol and cultured using unsterilized natural water from Xuanwu Lake (Nanjing, China) supplemented with 2.5 mM phenol, respectively, and the phenol concentration was measured by HPLC using the above system at intervals of 12 h. *Penicillium chrysogenum* CBS 306.48 was cultured in liquid Czapek-Dox medium (3.0 g/L NaNO3, 30.0 g/L Sucrose, 1.3 g/L K2HPO4•3H2O, 0.5 g/L MgSO4•7H2O, 0.5 g/L KCl, 0.01 g/L FeSO4•7H2O, 0.005 g/L CuSO4•5H2O, 0.01 g/L ZnSO4•7H2O) before co-culture at 150 rpm at 25°C for 3 days. The fungi were washed repeatedly with MSM followed by dilution to 0.02 g/ml wet weight and finally added to 100 ml MSM with the 1 ml diluent. The determination of glucose concentration using the DNS colorimetric method was in accordance with a previously reported procedure [19] employing an ultraviolet spectrophotometer (Thermo, USA).

To obtain and compare the phenol degradation rates in different conditions discussed above, a nonlinear equation named the Richards model was used[20], which can be described as follows:

$$S=S_{0}\left\{1-{\left\{1+\left(m-1\right)∙e^{m}∙exp[\frac{\mu_{m}}{s_{0}}∙m^{\frac{m}{m-1}}∙\left(\lambda -t\right)]\right\}}^{\frac{1}{1-m}}\right\}$$

### Tests of phenol degradation enzymes and genes

The enzymes involved in phenol degradation in the strain TIBETAN4, including phenol hydroxylase (PH), catechol 1,2-dioxygenase (C12O) and catechol 2,3-dioxygenase (C23O), and their genes were tested. The organism was cultured in 50 ml MSM supplemented with 5 mM phenol for 2 days and collected by centrifugation at 10,000 g for 10 min. The collected strain was washed with phosphate buffer saline(PBS, 0.27 g/L KH_2_PO_4_, 1.42 g/L Na_2_HPO_4_, 8.0 g/L NaCl, 0.2 g/L KCl, pH 7.4) 3–5 times. Finally, the strain was resuspended with 10 ml PBS. The collected cells were disintegrated by the ultrasonic wave, and the broken bacterial suspension underwent ultracentrifugation at low temperature (10,000 g, 15 min, 4°C); the supernatant was gathered in sterilized EP tubes followed by storage at 4°C for subsequent enzyme activity assays. The total protein concentration was analyzed for its crude enzyme extract as previously reported by Bradford et al[21], and bovine serum albumin was used as the standard. Determination of enzyme activity and specific enzyme activity was performed as described [1,22].

For tests of the phenol degradation gene, specific primer sequences were designed according to the published *Kocuria sp*. strain genome sequence and literature[23] (Table 1). Genome extraction, PCR reaction mixtures, and visualization of the product were performed as described above. The PCR parameter for PH/C12O/C23O was pre-denaturation at 94°C for 5 min, 35 cycles of 94°C for 35 s, 71°C/71°C/55°C for 45 s, and 72°C for 30 s, followed by a final elongation step at 72°C for 5 min. For the transcription of phenol degradation genes, the strains growing in MSM with 5.0 mM phenol were recorded as the Coercion Group, while the strains growing in MSM supplemented with 1.5% glucose were recorded as the Normal Control. The strains of both groups were collected at day 0, day 1, day 2 and day 3, and total RNA was extracted from each group using the RNA extraction kit (Sigma, USA) at various time intervals and reverse transcribed into cDNA using the total RNA reverse transcription kit (TSINGKE, China) according to the manufacturer’s instructions for subsequent q-PCR detection. The phenol degradation-related genes, PH and C12O genes, play a key role in phenol degradation based on the results of the enzyme activity analysis and the gene detection discussed above. The PCR primers of PH and C12O genes were also selected as specific q-PCR primers, and the 16s rDNA gene was selected as the reference (Table 2). A real-time fluorescence quantitative PCR (CFX96, BIO-RAD) was employed, and the reaction program was designed as follows: pre-denaturation at 95°C for 5 min, 40 cycles of 95°C for 10 s and 70°C for 30 s, followed by a final step at 95°C for 15 s, 70°C for 60 s, and 95°C for 15 s.

**Table 1:** Design of PCR/q-PCR primers for phenol degradation related genes including PH, C12O, and C23O.

Gene	Primer (5’-3’)	Tm (°C)	Product length
PH^a^	F: CCGCCAGATCATCGGGGACACGGACT	70	168
	R: TCGCCCACGCCCGAGTTGAGC		
C12O^a^	F: GTGCTGCCCACTCCCGCGACCCT	70	223
	R: TCTCGAAGTACCCGTTCTCGTCGGCCTT		
C23O^b^	F: AAGAGGCATGGGGGCGCACCGGTTCGATCA	55	380
	R: CCAGCAAACACCTCGTTGCGGTTGCC		
16s rDNA^a^	F: GCGGTTTGTCGCGTCTGCTGTG	70	149
	R: TGCCTTCGCCATCGGTGTTCCT		

a. Designed according to the reported genome of *Kocuria sp*. from GenBank

b. Data from Táncsics, Szoboszlay et al. 2012[23].

**Table 2:** The tests of carbohydrate utilization and enzyme activity of the strain TIBETAN4.

Carbohydrate utilization	TIBETAN4	PDM-7^a^	Features	TIBETAN4	PDM-7^a^
Xylose	**+**	**+**	ONPG hydrolysis	-	+
Adanitol	**+**	**-**	Lysine decarboxylases	**-**	+
Glucose	**+**	**-**	Ornithine decarboxylases	-	+
Rhamnose	**+**	**+**	Urease	**-**	**-**
Arabinose	**-**	**+**	Phenylalanine deamination	**-**	+
Lactose	**+**	**-**	Methyl red	**-**	-
Raffinose	**-**	**-**	Nitrate reduction	**+**	**+**
Melibiose	**+**	**-**	Tryptophan deamination (Indole)	**+**	**-**
Saccharose	**-**	**-**	Malonate	**-**	**-**
Trehalose	**+**	**-**	Hydrogen sulfide production	**-**	**-**
Celliobiose	**-**	**-**	Citrate utilization	**+**	**-**
Oxidase	**-**	**-**	Vogesproskaur’test (VP)	**-**	**-**
Catalase	**+**	ND	Esculin hydrolysis	**+**	**-**
Melanin	**-**	ND			
D-fructose	**+**	ND			

1) +, >90% strain was positive; d, 11%-89% strain was positive; -,> 90% strain was negative; ND, no determination; 2) a. Data from Reddy, Yusoff et al. 2005[24].

### Statistical analysis

All experiments were conducted in three replicates, and all results are presented as the mean ± sd. The software SPSS 23.0(IBM, USA) was used for statistical analysis, and unpaired Student’s t-test was used to detect the differences between groups with *, ** and *** indicating 0.01≤p<0.05, 0.001≤p<0.01 and p<0.001, respectively.

## Results

### Separation and screening

In the preliminary screening, 9 isolates from 13 different sample sites, named TIBETAN1-9, could grow well in the MSM with 10 mM phenol as the sole carbon source (Part A of S2 Fig and S1 Table). Taking into account that the inevitable mix of other trace carbon sources from soils could lead to the growth of the strain, a secondary screening was conducted with the 9 isolates to confirm the capacity of phenol degradation. The results indicated that TIBETAN4 had the strongest phenol-degrading ability compared with the isolates TIBETAN1-3 and TIBETAN5-7 (Part B of S2 Fig). The strain TIBETAN4 was selected for subsequent experiments.

### Identification of TIBETAN4

**Fig 1:**
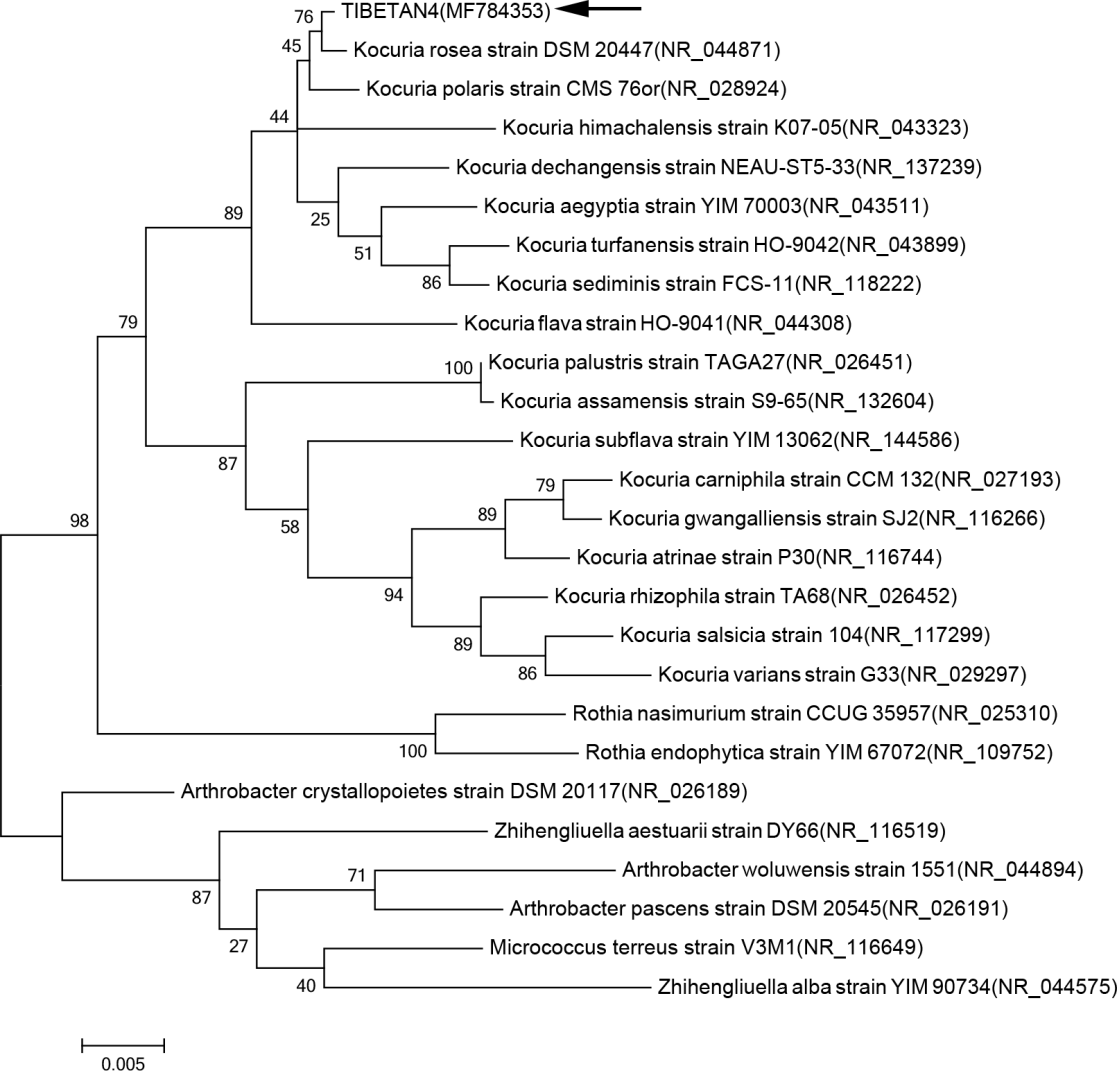
Phylogenetic tree based on Maximum Likelihood method. Phylogenetic tree for TIBETAN4 and its related species generated from Maximum Likelihood (ML) analysis of 16s rRNA gene sequences. Bootstrap support of branches indicated on the node was obtained using 1,000 replicates. Branch lengths are indicated as 0.005 substitutions per position according to the scale bar underneath the tree.

The 16s rRNA of strain TIBETAN4 was amplified and collected using PCR, and the aligned dataset for 16s rRNA was 1,386 bp long. The GenBank accession no. for TIBETAN4 16s rRNA was MF784353. Blastn results showed that the 16s rRNA of strain TIBETAN4 has high sequence identity with *Kocuria sp*. The sequence identity of the 16s rRNA gene with the *Kocuria rosea* strain DSM 20447 appeared with the highest sequence identity up to 99.8%. 16s rRNA multi-genomic sequence were aligned using ClustalW method and phylogenetic tree was made using maximum likelihood method (ML) in which the 16s rRNA gene sequence of strain TIBETAN4 clustered with that of *Kocuria rosea* strain DSM 20447 (Fig 1). The neighbor-joining (NJ) and maximum parsimony (MP) analyses were also conducted and yielded similar topologies (S3 and S4 Figs).

**Fig 2:**
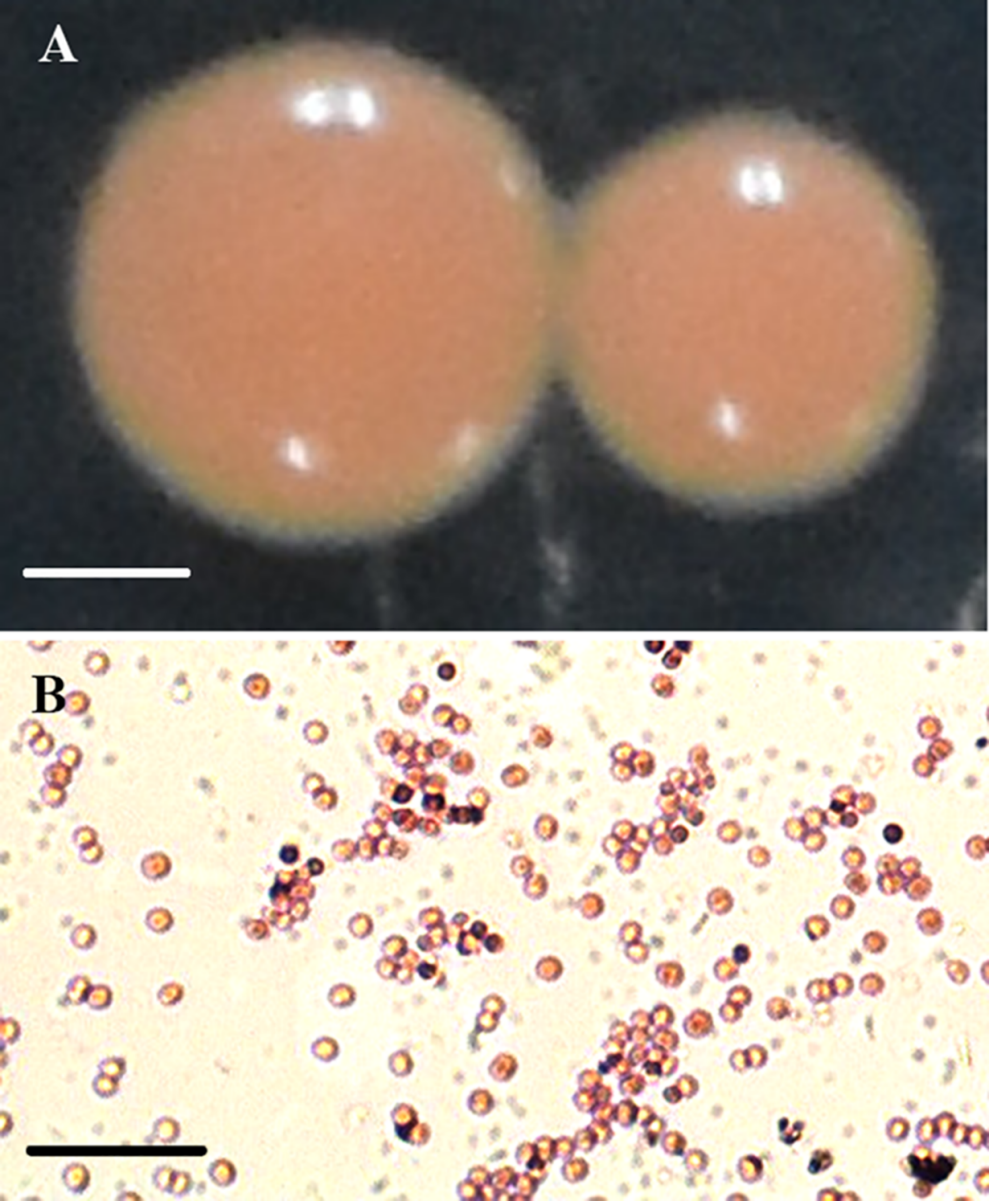
Morphology of strain TIBETAN4. (A) Colonies of strain TIBETAN4 grown on LB plates for 5 days, Scale bars = 0.5 mm; (B) Complex red single staining of strain TIBETAN4 Scale bars = 10 μm.

For microscopic studies, strain TIBETAN4 is a gram-positive bacterium without significantly sporty unathletic. When cultured on the LB agar plates, the colonies were round and convex with a smooth surface, complete edge and orange color (Fig 2). For the biochemical tests, the enzyme activity of the strain TIBETAN4 showed that the nitrate reduction, Tryptophan deamination (Indole), Citrate utilization and Esculin hydrolysis tests were positive while others were negative. The tests of carbohydrate utilization showed that the strain TIBETAN4 could use xylose, adonitol, glucose, rhamnose, lactose, melibiose, trehalose, catalase, and D-fructose but not arabinose, raffinose, saccharose, cellobiose, oxidase or melanin. The *Kocuria sp*. strain PDM-7 has the ability to degrade pollutants[24] and was listed in comparison with TIBETAN4 (Table 2).

### Tolerance for pH and NaCl

**Fig 3:**
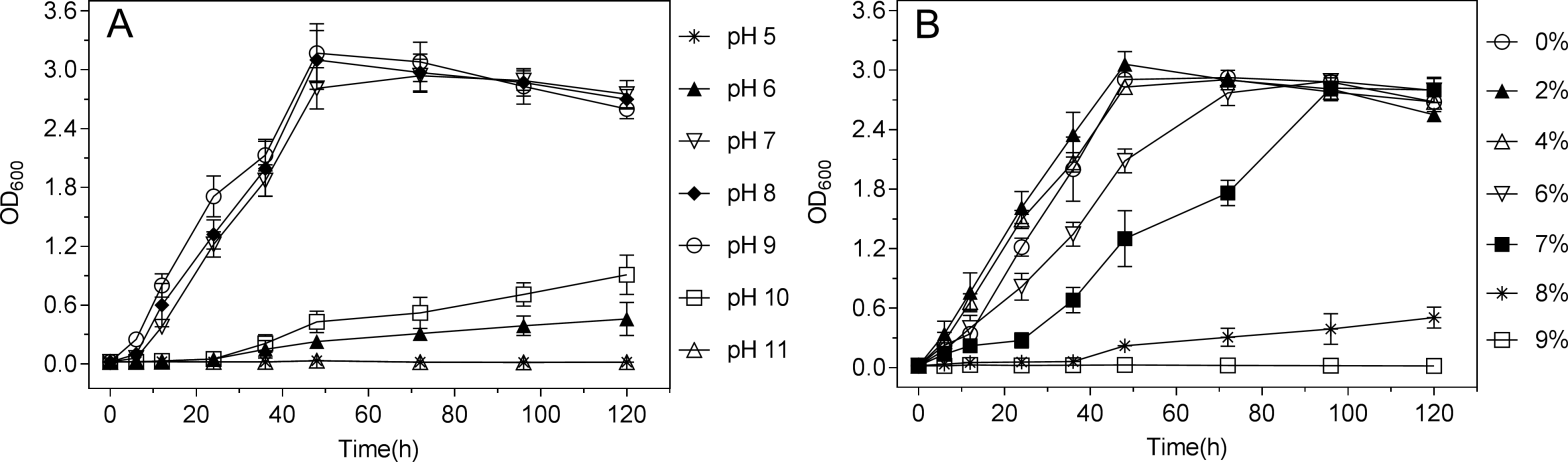
Growth curve of strain TIBETAN4 in LB. (A) Adjusted to different pH (5–11); (B) Adjusted to different concentrations of NaCl (0–9%). A, B. Cultured at 150 rpm, 25°C in the darkness.

The bacterium strain TIBETAN4 was cultured in LB liquid medium at pH 5–11 and 0–9% (0–1.54 M) NaCl, respectively, at 200 rpm, 25°C in the dark for 120 h. Samples were taken at specific intervals, and the OD_600_ value was measured. For tests of pH, TIBETAN4 showed a certain alkaline resistance with a good growth at pH 7–9 and plateaued after 48 h (Fig 3A). However, the growth of the strain was significantly inhibited as the pH continued to increase or decrease. When the pH was 6 or 10 and both of the isolates grew slowly, the strain grew faster at pH 10 than at 6, which further demonstrated that the strain TIBETAN4 was tolerant to alkali yet intolerant to acidic conditions. There was no growth and reproduction of TIBETAN4 at the pH 5 or 11. For studies of NaCl concentration, TIBETAN4 also had tolerance to NaCl and grew normally under the 0–4% NaCl (Fig 3B). With the continuous increase in NaCl concentration, the growth of the strain was progressively restrained, and the time reaching the plateau was prolonged. The organism could tolerate up to 8% NaCl. When the concentration reached 9%, the strain stopped growing. In addition, the growth of the isolate at pH 9 or 2% NaCl was superior to that at pH 7 or 0% NaCl, meaning that the strain was basophilic as well as acidophilic.

### Metabolism of phenol

**Fig 4:**
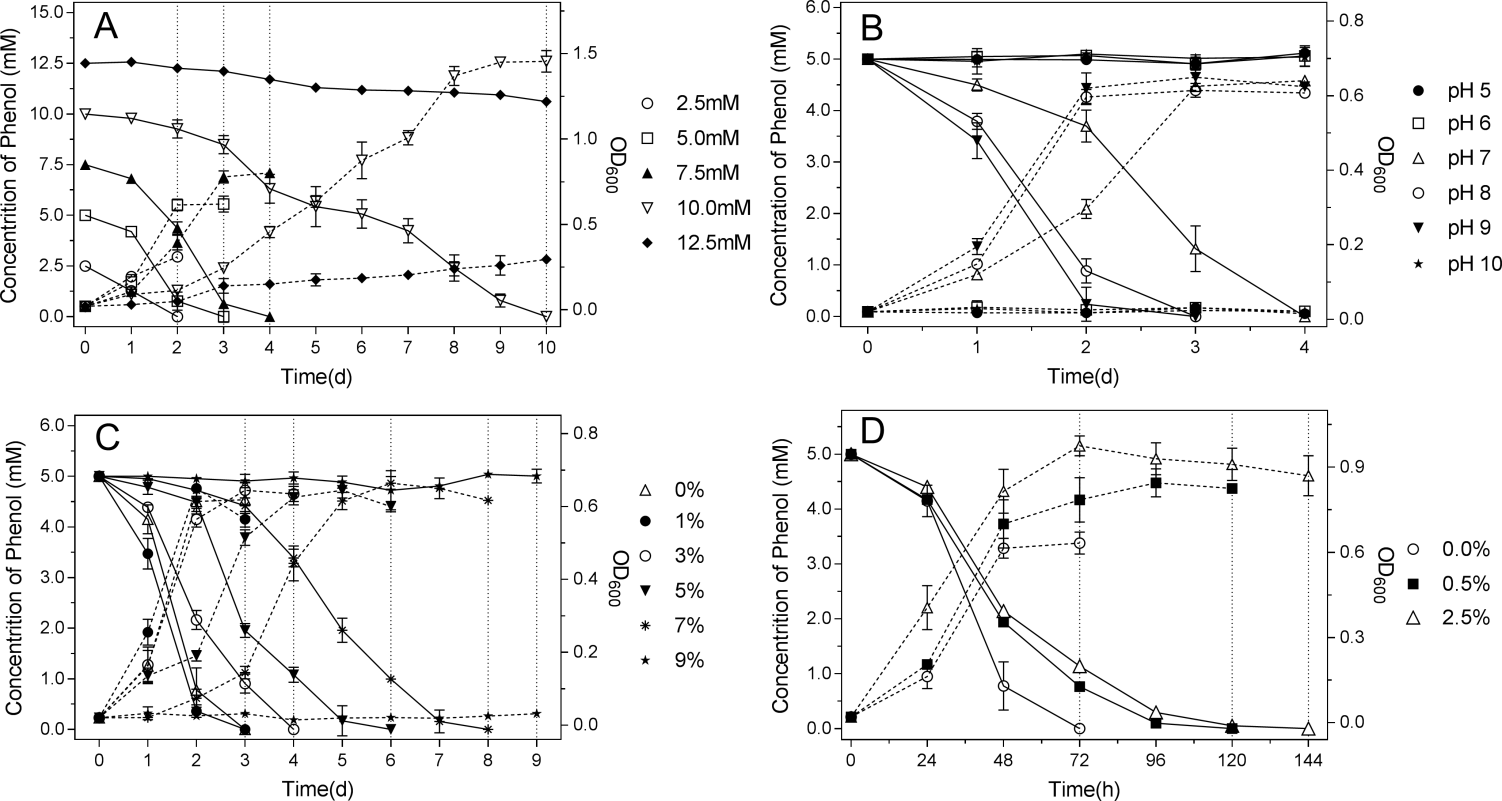
Phenol degradation ability and growth curve of strain TIBETAN4 in MSM. (A) Added with different concentrations of phenol (2.5–12.5 mM); (B) Added with 5.0 mM phenol and adjusted to different pH values (5–10); (C) Added with 5.0 mM phenol and adjusted to different concentrations of NaCl (0–9%); (D) Added with 5.0 mM phenol and different concentration of glucose (0–2.5%). A-D. Cultured at 150 rpm, 25°C in the darkness. The solid line shows the change in the phenol concentration, and the dotted line shows the change in the OD value of the strain.

TIBETAN4 showed high phenol-degrading activity with a high tolerance of phenol up to 12.5 mM. The strain could degrade 5.0 mM phenol within 3 days and 7.5 mM phenol within 4 days with phenol as the sole carbon source. However, the maximum phenol degradation rate under 7.5 mM initial phenol concentrations was approximately 350.089 mg l^-1^ day^-1^, which was higher than under 5.0 mM with approximately 325.621 mg l^-1^ day^-1^ according to the data calculated by the Richards Model (Table 3). The time for phenol degradation was lengthened when the phenol concentration was increased to 10.0 mM, and it took 10 days to completely degrade the phenol. Only a trace amount of phenol was degraded by the 10th day with the phenol concentration at 12.5 mM, and the biomass increased slightly, suggesting that the growth of TIBETAN4 at the phenol concentration was significantly inhibited, which explains the non-degradation of phenol (Fig 4A).

**Table 3:** Comparison of the maximum rate of phenol degradation under different conditions, including the initial concentration of phenol, salt content, pH and glucose content, calculated based on Richards model.

Condition		μ_m_ (mg l^-1^ day^-1^)	Condition		μ_m_ (mg l^-1^ day^-1^)
			Salt content	0.0%	322.797
The initial concentration of phenol	2.5 mM (235.275 mg l^-1^)	134.577		1.0%	346.325
	5.0 mM (470.550 mg l^-1^)	325.621		3.0%	231.511
	7.5 mM (705.825 mg l^-1^)	350.089		5.0%	207.983
	10.0 mM (941.100 mg l^-1^)	155.282		7.0%	125.166
pH	7	219.276	Glucose content	0.0%	324.680
	8	324.680		0.5%	318.092
	9	309.622		2.5%	217.394

The strain maintained efficient phenol degradation activity under weakly alkaline conditions, and compared with the degradation of 5.0 mM phenol within 4 days at pH 7, the strain could degrade the same concentration of phenol within 3 days at pH 8 and 9 while reaching the highest degradation rate at approximately 324.680 mg l^-1^ day^-1^ at pH 8 (Fig 4B and Table 3). When the pH value was 5 or 6, the strain did not have phenol degradation activity, which may result from its loss of growth activity, and the growth curve also indicated that the strain basically stopped growing. However, the strain lost phenol-degrading activity and ceased its development when the pH rose to 10 or dropped to 5 and 6. TIBETAN4 maintained high efficiency of phenol degradation under the high NaCl concentration, and 6 days when combined with 5% NaCl, while with the same concentration of phenol combined with 7% NaCl, 5.0 mM phenol could be degraded completely within 7 days. The strain forfeited phenol-degrading activity at 9% NaCl with its biomass significantly inhibited. It is noteworthy that the conditions of 1% NaCl and pH 9, compared with 0% NaCl and pH 7, showed an increase in phenol-degrading ability (Fig 4C and Table 3), which may be related to their biomass, indicating that the isolate was halophilic and alkaline, which was consistent with the results of strain tolerance discussed above.

The phenol degradation activity of TIBETAN4 was inhibited slightly when added with 0.5% or 2.5% glucose, and with the increase in glucose levels, the inhibitory effect of phenol degradation activity in the strain increased simultaneously. The isolate still maintained a high activity of phenol degradation and could totally degrade 5.0 mM phenol in 5–6 d when supplied with 0.5% or 2.5% glucose and within 3 d without glucose (Fig 4D). The maximum phenol-degradation rate of TIBETAN4 without added glucose was approximately 324.680 mg l^-1^ day^-1^ which was more effective than that with 0.5% or 2.5% added glucose with 318.092 mg l^-1^ day^-1^ and 217.394 mg l^-1^ day^-1^, respectively (Table 3). The biomass of TIBETAN4 with the concurrent addition of glucose and phenol was inhibited compared to that with only glucose added, which may be explained by the cytotoxic effect of phenol on the strain (Part A of S5 Fig). In addition, the utilization of phenol and glucose was in progress at the same time (Part B of S5 Fig). The strain still maintained high efficiency of phenol degradation and digested 2.5 mM of phenol within the 60 h when the *Penicillium* strain that was widely distributed in the environment was mixed in the medium (Part C of S5 Fig) and even natural lake water was directly used to make medium (Part D of S5 Fig).

### Analysis of enzymes and genes

**Fig 5:**
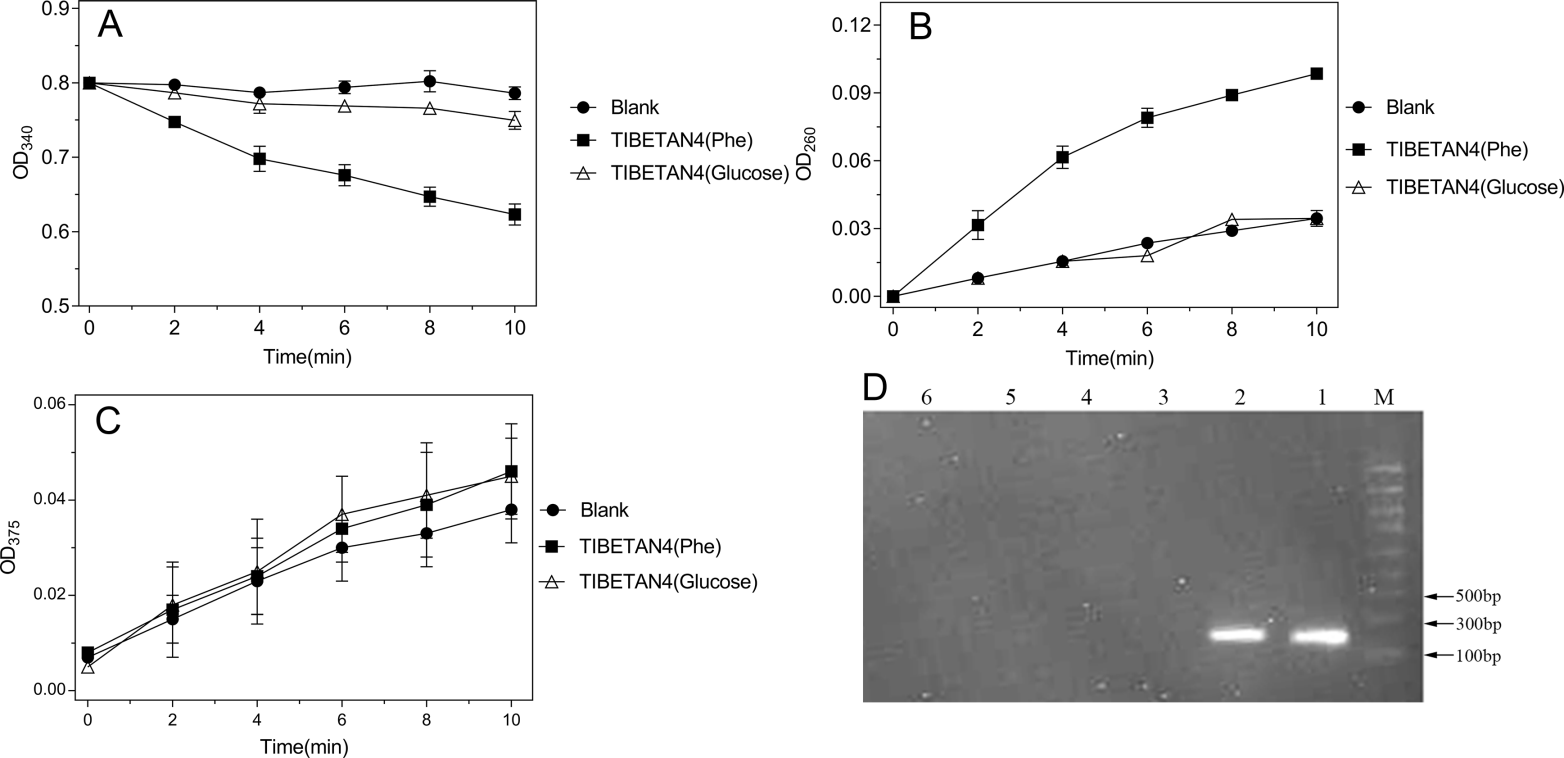
Tests of phenol degradation-related enzymes and genes. The activity of enzymes related to phenol degradation from TIBETAN4 cultured in MSM added with 5 mM phenol or 2.5% glucose as the sole carbon source were detected once every two minutes for a total of 10 minutes, and PBS was used as blank control. (A) The activity curve of phenol hydroxylase was 0.051±0.008U/mg; (B) The activity curve of catechol 1,2-dioxygenase was 0.213±0.014U/mg; (C) The activity curve of catechol 2,3-dioxygenase was not detected; (D) The genes of phenol degradation related enzymes, including PH and C12O, in the genome of TIBETAN4 were detected. Descriptions were in “Target gene-Template” format as follows: 1 PH-TIBETAN4; 2 C120-TIBETAN4; 3 C23O-TIBETAN4; 4 PH-H_2_O; 5 C120- H_2_O; 6 C23O- H_2_O, and a 5,000 Marker was used.

TIBETAN4 showed strong phenol hydroxylase and catechol 1,2-dioxygenase activity, without catechol 2,3-dioxygenase activity, after induction by phenol, which suggested the isolate degraded phenol through the reaction of phenol hydroxylase and catechol 1,2-dioxygenase, also known as the ortho-degradation pathway, instead of the meta-degradation pathway (Fig 5A, 5B & 5C). The specific activities of phenol hydroxylase and catechol 1,2-dioxygenase were 0.051±0.008 U/mg and 0.213±0.014 U/mg, respectively.

Specific primers of the key genes of phenol degradation, including phenol hydroxylase, catechol 1,2-dioxygenase, and catechol 2,3-dioxygenase, were designed to amplify these genes usingthe genomic of TIBETAN4as a template (Fig 5D).The specific amplification bands of phenol hydroxylase and the catechol 1,2-dioxygenase gene were obtained without the catechol 2,3-dioxygenase gene, and no band was found in the negative control group where the genomic DNAwas replaced with ddH_2_O. This indicated that the strain has the potential to degrade phenol through ortho-ring opening instead of meta-ring opening, which was consistent with the results of the enzyme activity assay.

**Fig 6:**
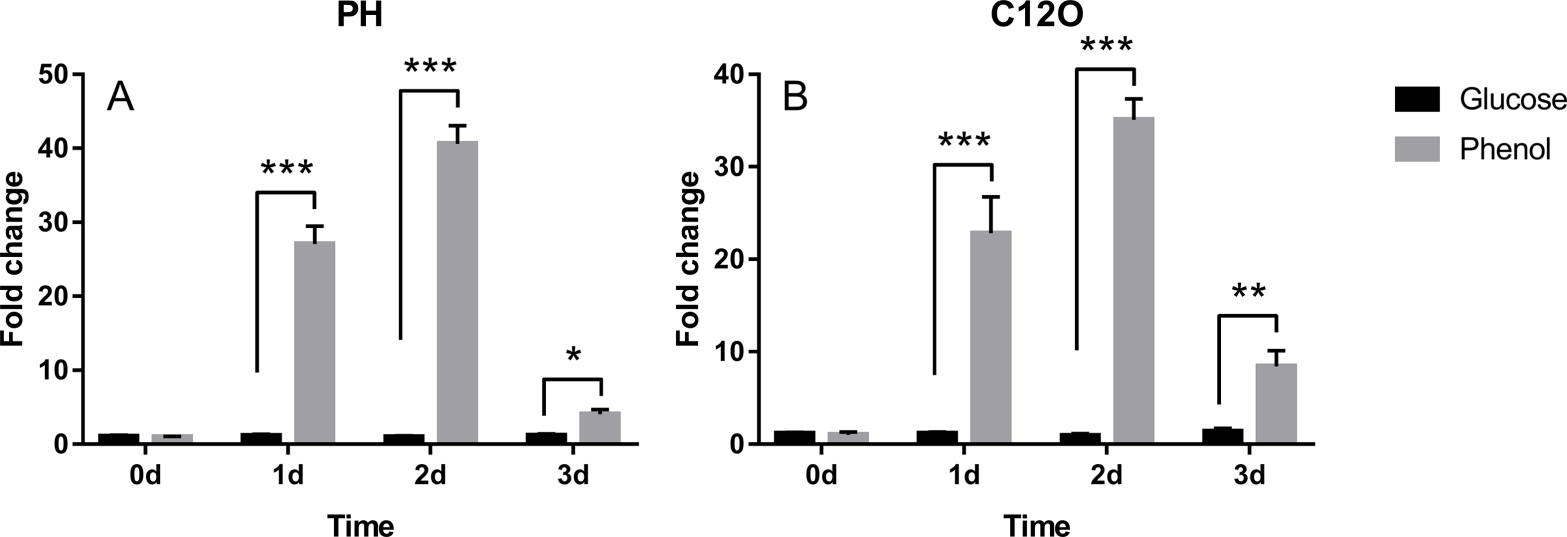
Q-PCR analysis of PH and C12O gene of TIBETAN4. The results showed a significant increase under phenol stress compared with that under glucose stimulation and were most significant on the second day, indicating that phenol can effectively promote the transcription of PH and C12O genes.

The results of q-PCR showed that the transcript levels of gene of the phenol hydroxylase and catechol 1,2-dioxygenase in the strain TIBETAN4 under phenolic stress were significantly increased on the first, second, and third day compared with non-stress (Fig 6). All of them had the highest level of transcription on the second day, while on the third day, although the transcription level of the stress group was still significantly higher than that of the normal control group, the transcription level was significantly lower than that on the second day, which may result from the complete phenol degradation by the strain (data not show), which proved once again that TIBETAN4 has the ability to degrade phenol by the ortho cleavage pathway.

## Discussion

Environments including polluted water and soil, which always show strong salinization or acidity, tend to be extremely more complex because of the existence of a large number of organic pollutants and high concentrations of various types of carbon sources[25]. These complex physicochemical properties led to the strain that proved to efficiently degrade contaminants under laboratory conditions and performed poorly under natural environmental conditions, even losing its original pollutant degradation ability, with a great possibility of inhibiting the growth or the enzymatic activity of the key degradation processes of the strain. To determine the environmental remediation isolates that have real application value, it is necessary to detect the tolerance of screened strains in various conditions, such as various pH levels and NaCl concentrations. In this study, the strain TIBETAN4 showed strong tolerance to an adverse environment, which may result from the long-term adaptation of saline lakes to the harsh environment. There had been many microorganisms showing strong salt and alkali resistance isolated from the saline lakes[26,27] and salt tolerant plants living in or around the saline lake[28,29]. The acquisition and retention of characteristics of adaptability to various stresses by microbes in extreme environments are essentially found in the adaptive evolution process of the anti-stress function at the gene level, which means that it can be transmitted to descendants through asexual reproduction to maintain the reproduction of its population[30]. Interestingly, Patel A et al. found that a phenol degrading yeast, *Rhodosporidium kratochvilovae* HIMPA1, could transform phenol into lipid granules, which could help to enhance its resistance to cold and salt when stored in the body [31]. Thus, it is not difficult to guess that for a phenol degradation strain in the natural environment, phenol may not only serve as an energy source but also play an important role in other aspects of the survival of the strain. Unlike the strain CLONA2[32], which is strongly inhibited by glucose or other carbon sources, the addition of glucose for TIBETAN4 only slightly prolonged the time required for complete degradation of phenol, and such a characteristic greatly expands the application scope of the isolate. The delay may be caused by the simultaneous use of glucose and phenol by cells, which resulted in reduced dependence of phenol as a source of energy. This could be one of the main reasons why TIBETAN4 needs more time to degrade the same concentration of phenol in natural lake water than that in MSM considering other complex carbon sources in natural lakes.

Most of the reported phenol degradation strains are derived from industrial wastewater and soil, which are always greatly influenced by human factors. Nevertheless, the unfortunate reality is that there are few reports of phenol-degrading microorganisms from extreme natural environments, which is helpful not only for the discovery of phenol-degrading microorganisms with excellent characteristics but also for studying the factors affecting the formation of phenol degradation activity and the interactions between strains and their surrounding environment. In fact, many studies have shown that the compounds derived from plants such as polychlorinated biphenyls (PCBs) and polycyclic aromatic hydrocarbons (PAHs)[33,34] can affect microbial biodegradation pathways. This phenomenon is called the "secondary complex hypothesis", which is also an important element for some plants to survive in extreme environments[35] through the association of bioremediation or biodegradation microbes around the plant rhizosphere. Furthermore, it has been reported that in the polluted environment, the composition of rhizosphere microbial communities could be changed by the synthesis and secretion of rhizospheric secretion and be more efficient in pollutant degradation[36]. In addition, S Fraraccio et al. found that the growth and degradation ability of microbial cis-1,2-dichloroethylene were promoted using the monocyclic aromatic hydrocarbons obtained from plant secondary metabolites, which further validates the "secondary complex hypothesis"[37]. Therefore, it is of great value to explore the strains that degrade pollutants in the natural environment and explore their interactions with the surrounding environment on the basis of exploring their phenol degradability. This is also the direction of our efforts for strain TIBETAN4.

Most microorganisms usually degrade phenol through the aerobic pathway in which the phenol hydroxylase catalyzes phenol to catechol, which is the first and key step of phenol degradation and often directly affects the degradation rate of phenol. Then, this pathway can be divided into the ortho-pathway and meta-pathway, which are two separate metabolic systems according to the opening mode of the aromatic ring[38,39]. Interestingly, different kinds and even concentrations of aromatic hydrocarbons can activate different metabolic pathways of some certain microorganisms, with *Pseudomonas cepacia* as an example in which salicylate can only activate the ortho-pathway while benzoate can activate both the ortho-pathway and meta-pathway[40,41]. B. Cao et al. also reported a *Pseudomonas putida* strain whose only ortho-pathway was activated in the low concentration of benzoate (200–300 mg/l), and both kinds of pathways would be activated when grown in higher concentrations of benzoate[42].

According to the analysis of PCR amplification based on the specific primers and detection of enzyme activity, we found that TIBETAN4 after induction by phenol showed activity of the enzymes PH and C12O at 0.06664 + 0.0052 U/mg and 0.28848 + 0.017 U/mg, respectively, while in absence of C23O. This was consistent with the results of Q-PCR, which further proved that TIBETAN4 degraded phenol in the ortho-pathway without the potential of meta-pathway. Catechol 1,2-dioxygenase and catechol 2,3-dioxygenase, which work as not only the second important enzymes in the phenol degradation process but also toward labeling of the phenol degradation pathway, are always used to determine the phenol degradation pathway of different organisms[43,44]. It was reported that the biodegradable efficiency of the meta-pathway is often higher than that of the ortho-pathway on the basis of enzyme activity analysis[45]. However, in the light of the combination of database queries, including PUBMED (https://www.ncbi.nlm.nih.gov/pubmed), JGI (https://genome.jgi.doe.gov/portal/) and KEGG (http://www.kegg.jp/), it is an interesting finding that fungi always lack catechol 2,3-dioxygenase genes even with the ability to degrade phenol, indicating that these fungi degraded phenol using the ortho-pathway mainly under the same circumstances in TIBETAN4. This suggests that the ortho degradation pathway may have unexplored potential advantages that could lead to a choice for the microbes in the process of evolution.

In recent years, new environmental pollutant degradation methods based on biodegradation have been introduced. Methods combining the physical, chemical and biological degradation together to make up for the disadvantages of each by giving full play to their advantages have been receiving growing attention because they can be much more efficient, energy-saving, environmentally friendly, etc.[46]. Xie M et al. found that a mixture of different bacteria, among which a single strain presented no degradation ability because of an incomplete degradation pathway of aromatics, could realize the degradation of aromatic pollutants through the mutual complement and combination of the endemic abilities of various species[47]. Therefore, it is an effective strategy to combine the characteristics of various strains and design bacterial additives to repair the environment. TIBETAN4 maintained a high efficiency of phenol degradation in a both complex water environment and an environment in which *Penicillium* strains coexist, suggesting the application value of this strain and the feasibility of producing the bacteria additives.

## Supporting information

S1 Fig Map of the sampling site: Qinghai Lake. The sample plot was developed using ArcGIS (version 10.2.0) and based on the 2010 China Geographic Position Map.
(TIF)

S2 Fig Distribution of sampling point and comparison of phenol degradation ability of strains. A. Distribution of sampling site, the sample plot was developed using ArcGIS (version 10.2.0) and based on the 2010 China Geographic Position Map. B. Phenol degradation of strain TIBETAN1-9 in MSM added with 3 mM phenol at 150 rpm, 25°C for 3 days.
(TIF)

S3 Fig Phylogenetic tree based on Neighbor-Joining method. Phylogenetic tree for TIBETAN4 and its related species generated from Neighbor-Joining (NJ) analysis of 16s rRNA gene sequences. Bootstrap support of branches indicated on the node was obtained using 1,000 replicates. Branch lengths are indicated as 0.005 substitutions per positions according to the scale bar underneath the tree.
(TIF)

S4 Fig Phylogenetic tree based on Maximum Parsimony method. Phylogenetic tree for TIBETAN4 and its related species generated from Maximum Parsimony (MP) analysis of 16s rRNA gene sequences. Bootstrap support of branches indicated on the node was obtained using 1,000 replicates.
(TIF)

S5 Fig Phenol degradation and strain growth curve under different conditions. A. The growth curve of strain TIBETAN4 in MSM added with glucose(0.5%,2.5%), phenol(5 mM) and a mixture of phenol(5 mM) and glucose(0.5%,2.5%), respectively; B. The degradation of glucose and phenol by TIBETAN4 in MSM added together with 5 mg/ml glucose and 5mM phenol; C. The phenol degradation activity of TIBETAN4, CBS306.48 and a mixture of both respectively in MSM added with 5 mM phenol as the sole carbon source; D. The phenol degradation activity of TIBETAN4 cultured in MSM added with 5 mM phenol and non-sterilized lake water added with 5 mM phenol respectively and non-sterilized lake water added with 5 mM phenol without TIBETAN4 was used as blank control. A-D. Cultured at 150 rpm, 25°C in the dark.
(TIF)

S1 Table Characteristics of the sample sites. (DOCX)
