# Supplementary figures and images for: Degradation of phenol via ortho-pathway by Kocuria sp. strain TIBETAN4 isolated from the soils around Qinghai Lake in China
Source: PLoS One. 2018 Jun 27;13(6):e0199572. doi: 10.1371/journal.pone.0199572 (PMC6021097; doi:10.1371/journal.pone.0199572)

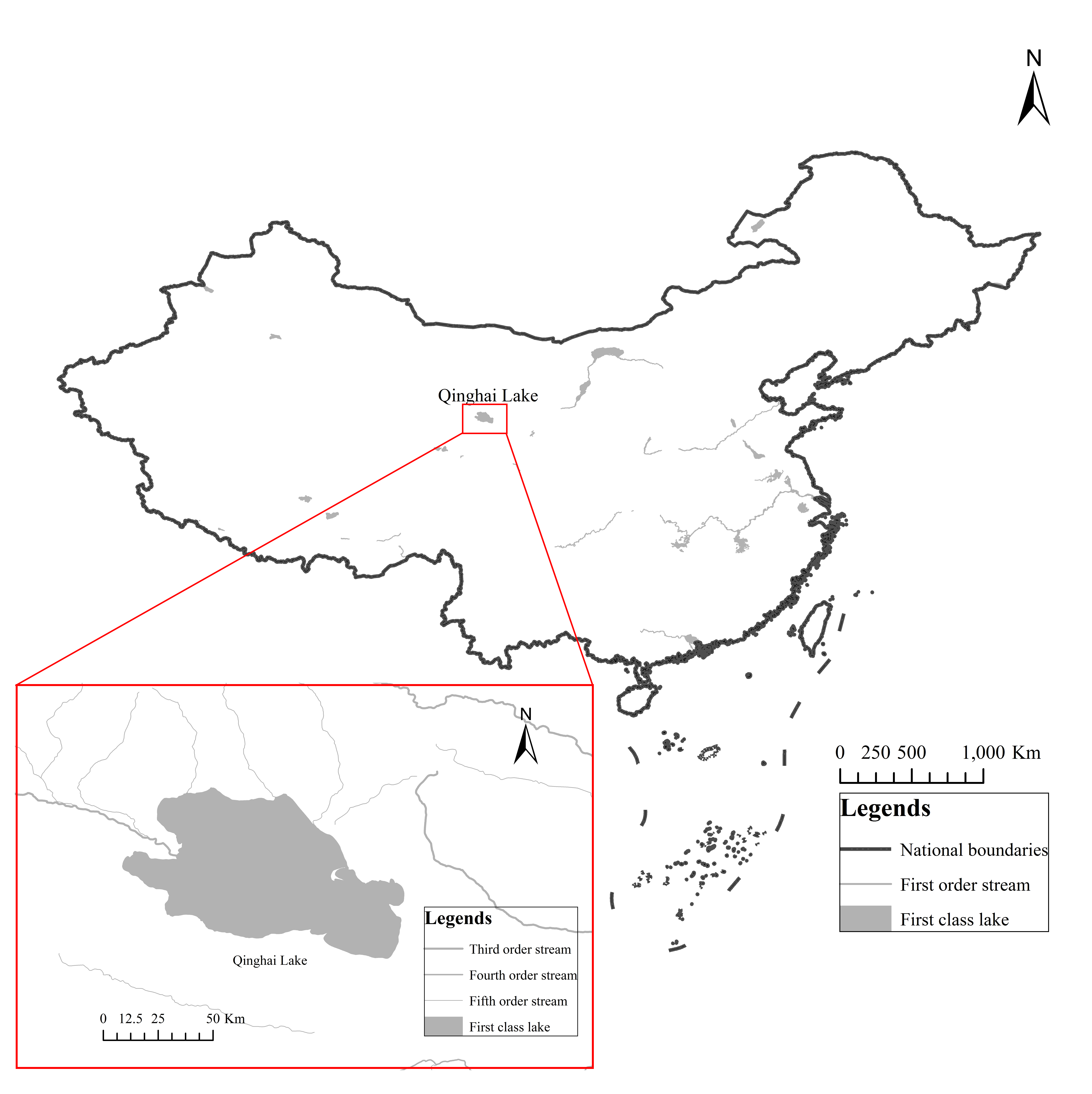

Supplement: S1 Fig — The sample plot was developed using ArcGIS (version 10.2.0) and based on the 2010 China Geographic Position Map. (TIF) [file pone.0199572.s001.tif]

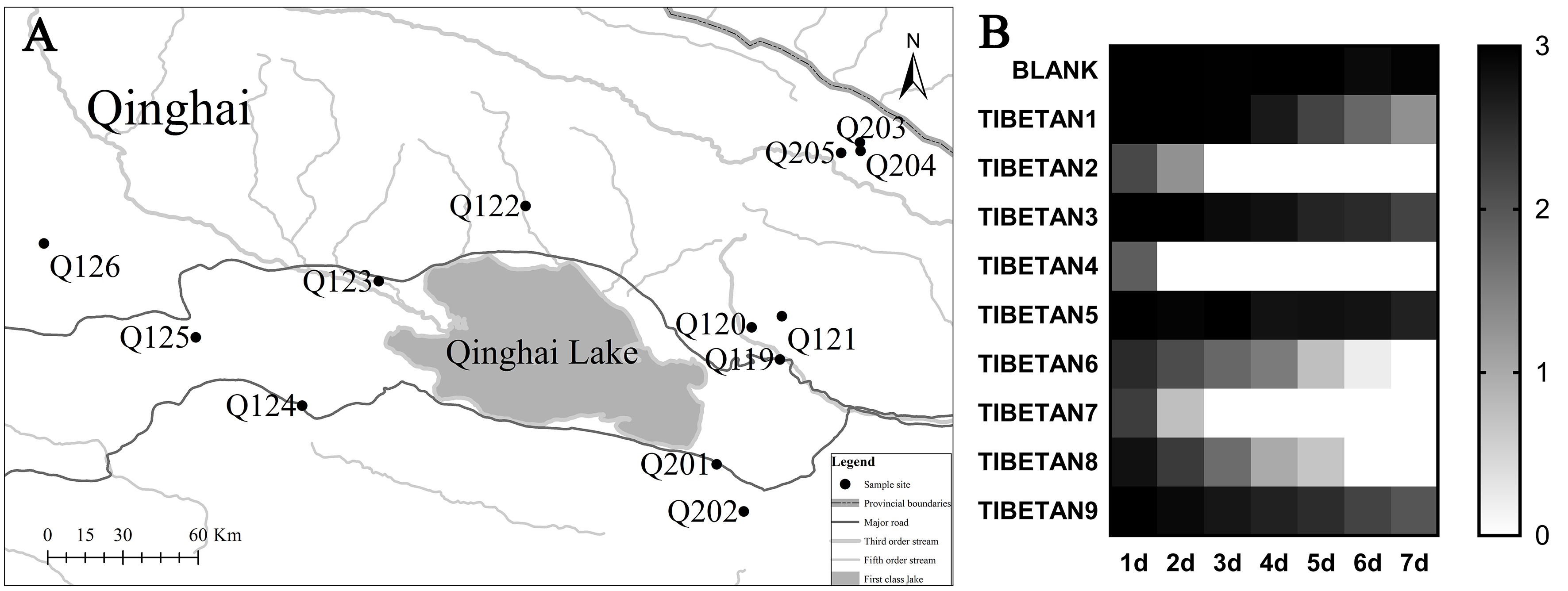

Supplement: S2 Fig — A. Distribution of sampling site, the sample plot was developed using ArcGIS (version 10.2.0) and based on the 2010 China Geographic Position Map. B. Phenol degradation of strain TIBETAN1-9 in MSM added with 3 mM phenol at 150 rpm, 25°C for 3 days. (TIF) [file pone.0199572.s002.tif]

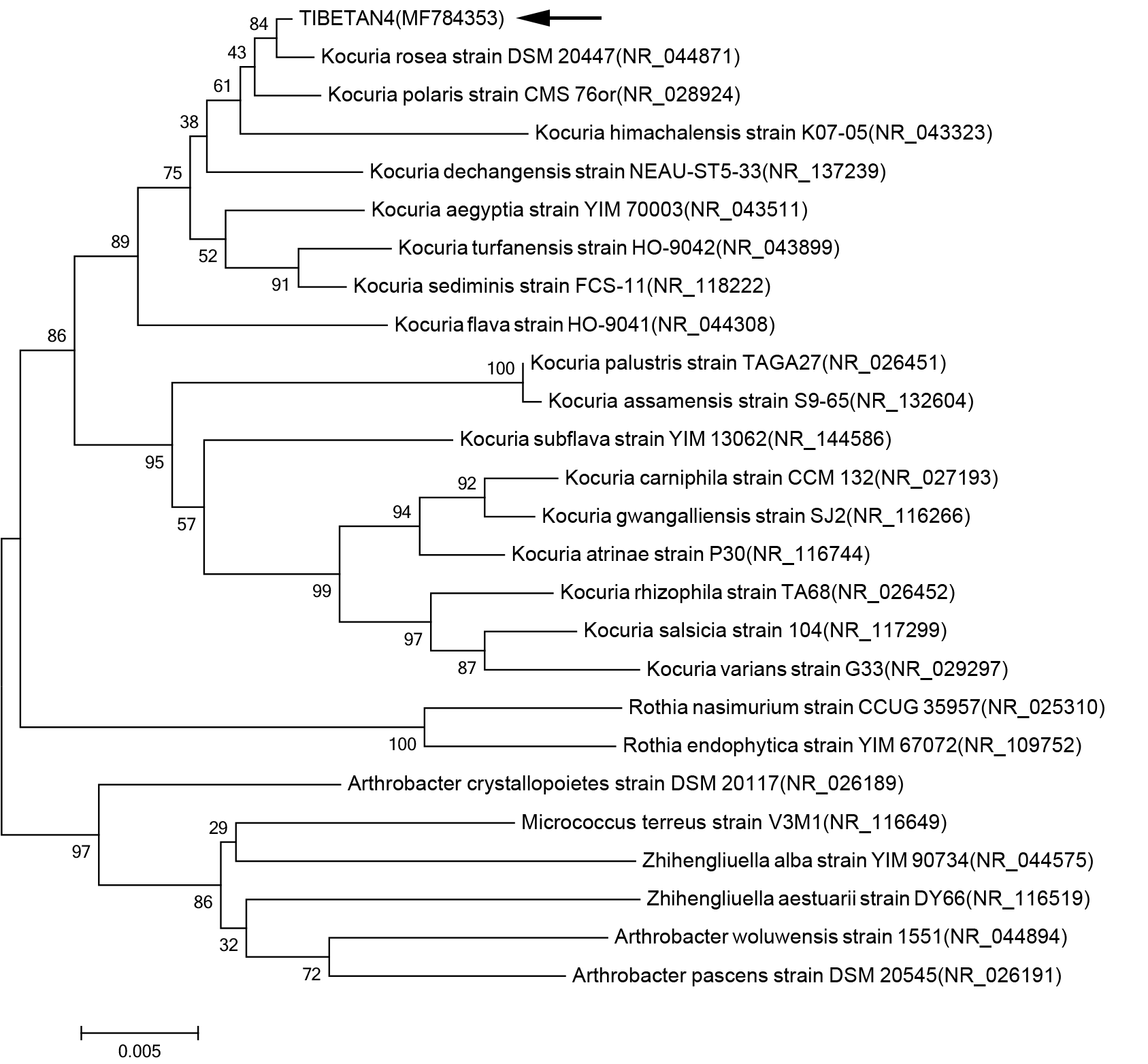

Supplement: S3 Fig — Phylogenetic tree for TIBETAN4 and its related species generated from Neighbor-Joining (NJ) analysis of 16s rRNA gene sequences. Bootstrap support of branches indicated on the node was obtained using 1,000 replicates. Branch lengths are indicated as 0.005 substitutions per positions according to the scale bar underneath the tree. (TIF) [file pone.0199572.s003.tif]

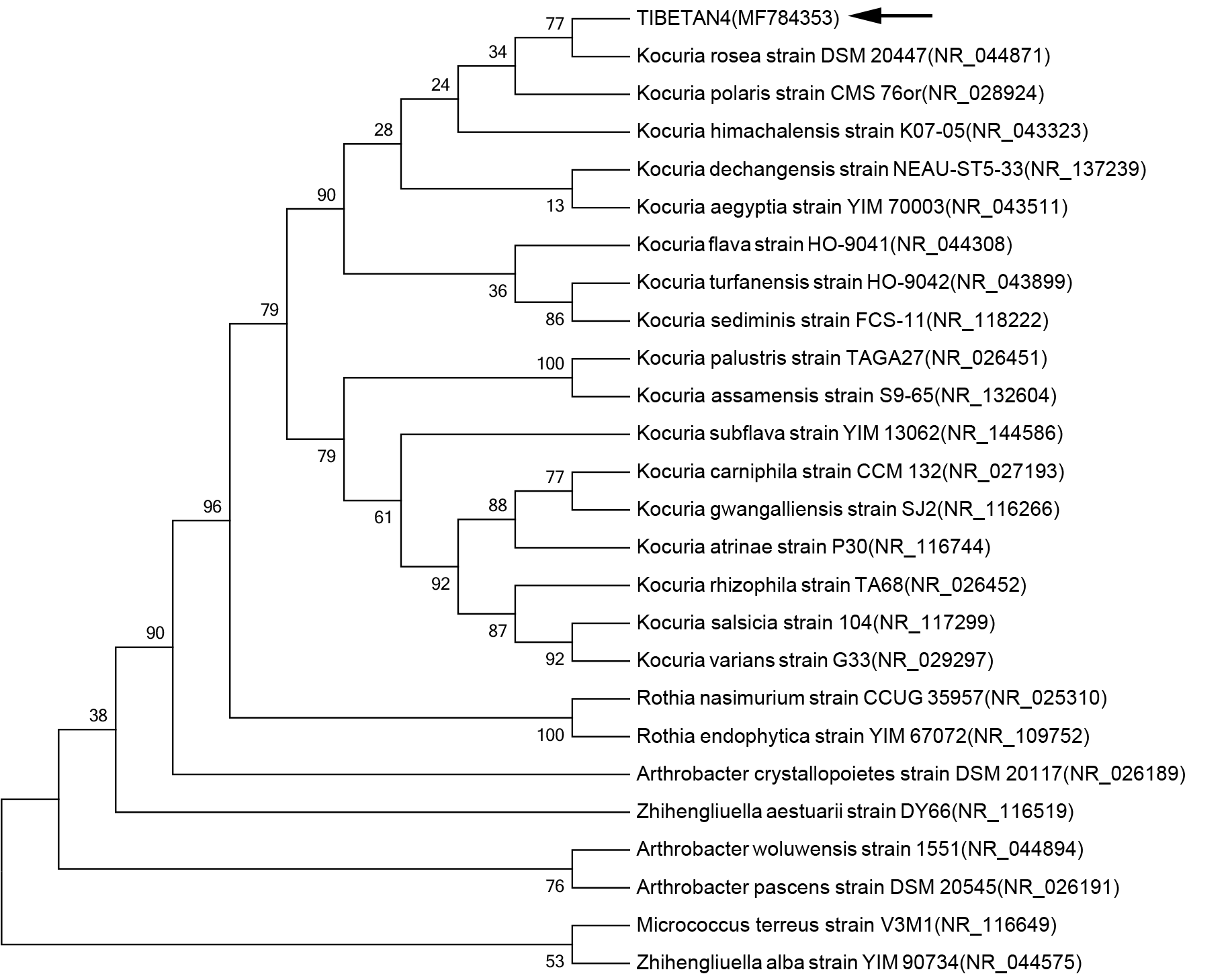

Supplement: S4 Fig — Phylogenetic tree for TIBETAN4 and its related species generated from Maximum Parsimony (MP) analysis of 16s rRNA gene sequences. Bootstrap support of branches indicated on the node was obtained using 1,000 replicates. (TIF) [file pone.0199572.s004.tif]

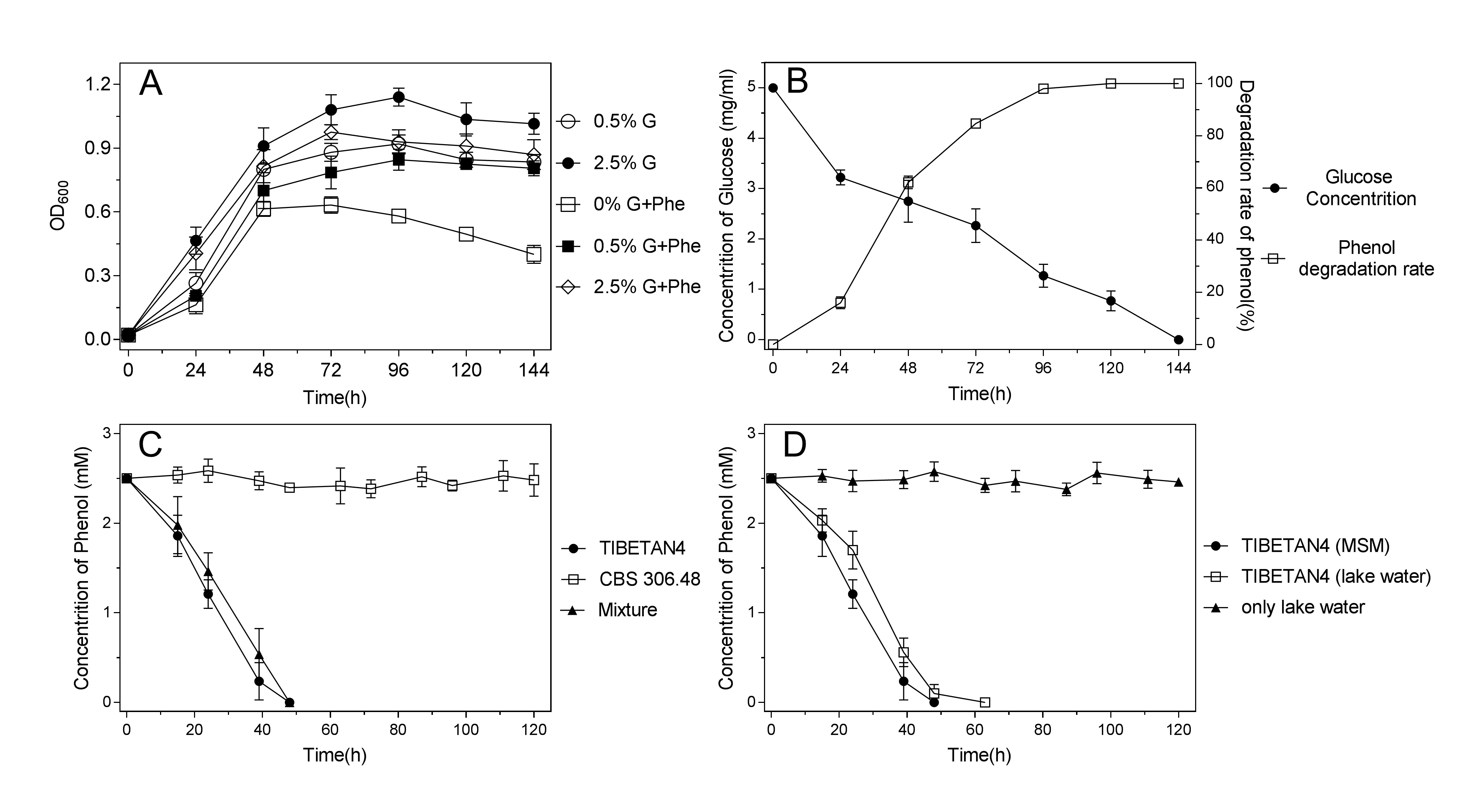

Supplement: S5 Fig — A. The growth curve of strain TIBETAN4 in MSM added with glucose(0.5%,2.5%), phenol(5 mM) and a mixture of phenol(5 mM) and glucose(0.5%,2.5%), respectively; B. The degradation of glucose and phenol by TIBETAN4 in MSM added together with 5 mg/ml glucose and 5mM phenol; C. The phenol degradation activity of TIBETAN4, CBS306.48 and a mixture of both respectively in MSM added with 5 mM phenol as the sole carbon source; D. The phenol degradation activity of TIBETAN4 cultured in MSM added with 5 mM phenol and non-sterilized lake water added with 5 mM phenol respectively and non-sterilized lake water added with 5 mM phenol without TIBETAN4 was used as blank control. A-D. Cultured at 150 rpm, 25°C in the dark. (TIF) [file pone.0199572.s005.tif]
