# Supplementary material for: Degradation of phenol via ortho-pathway by Kocuria sp. strain TIBETAN4 isolated from the soils around Qinghai Lake in China
Source: PLoS One. 2018 Jun 27;13(6):e0199572. doi: 10.1371/journal.pone.0199572 (PMC6021097; doi:10.1371/journal.pone.0199572)
Supplement: S1 Table — (DOCX) [file pone.0199572.s006.docx]

|  | pH | Temp.(°C) | Lat.(N) | Long.(E) | Alt.(m) | Soil type | Veg. type | Most common plant family | Grazing animal | Strain No. |
| --- | --- | --- | --- | --- | --- | --- | --- | --- | --- | --- |
| Q119 | 8.02±0.01 | 23 | 36°51′58.93″ | 101°01′16.29″ | 3,036 | Locss | Grassland | A.splendens;  S.breviflora | Sheep | TIBETAN4 |
| Q120 | 7.91±0.04 | 23 | 36°58′51.84″ | 100°55′11.04″ | 3,137 | Locss | Grassland | S.breviflora | Sheep | Not fround |
| Q121 | 7.60±0.01 | 23 | 37°01′16.49″ | 101°01′40.38″ | 3,193 | Locss | Meadow | K.humilis | Sheep | TIBETAN9 |
| Q122 | 7.74±0.19 | 21 | 37°24′59.10″ | 100°06′36.27″ | 3,400 | Locss to  Black Soil | Meadow | K.humilis | Sheep | TIBETAN1 |
| Q123 | 7.91±0.03 | 21 | 37°08′47.90″ | 99°35′02.43″ | 3,262 | Locss to  Black Soil | Grassland | S.sareptana | Sheep | Not found |
| Q124 | 8.13±0.06 | 27 | 36°42′02.98″ | 99°18′33.50′ | 3,198 | Locss | Grassland | A.splendens | Sheep | TIBETAN5 |
| Q125 | 8.08±0.03 | 27 | 36°56′43.71″ | 98°55′41.70″ | 3,711 | Locss | Grassland | S.breviflora | Sheep | Not fround |
| Q126 | 7.98±0.11 | 15 | 37°16′56.12″ | 98°23′02.90″ | 3,473 | Brown Locss | Grassland | S.breviflora | Sheep | Not fround |
| Q201 | 7.91±0.03 | 23 | 36°29′25.64″ | 100°47′39.33″ | 3,255 | Locss | Grassland | A.splendens | Sheep | TIBETAN6; TIBETAN7 |
| Q202 | 7.97±0.07 | 23 | 36°19′17.44″ | 100°53′29.10″ | 3,273 | Locss | Grassland | A.splendens | Sheep | TIBETAN8 |
| Q203 | 7.78±0.05 | 17 | 37°36′49.73″ | 101°18′35.66″ | 3,207 | Locss to  Black Soil | Meadow | E.nutans | Sheep | TIBETAN3 |
| Q204 | 7.79±0.09 | 17 | 37°38′38.42″ | 101°18′30.19″ | 3,322 | Locss to  Black Soil | Meadow | K.capillifolia | Sheep; Yak | Not found |
| Q205 | 8.13±0.06 | 17 | 37°36′23.69″ | 101°14′27.03″ | 3,213 | Locss to  Black Soil | Meadow | K.humips | Sheep; Yak | TIBETAN2 |
